# Loop-mediated isothermal amplification combined with a *Pyrococcus furiosus* argonaute system for the rapid detection of goose astrovirus

**DOI:** 10.1016/j.psj.2026.106397

**Published:** 2026-01-04

**Authors:** Fosheng Yang, Zhong Liu, Jianqiang Ye, Quan Xie, Chang Wu, Chengjun Jiang, Huangsheng Wu, Qianlang Gu, Deping Song, Fanfan Zhang

**Affiliations:** a Nanchang Key Laboratory of Novel Prevention and Control Agents for Animal Infectious Diseases, Institute of Animal Disease Prevention and Control, Jiangxi Agricultural University, College of Animal Science and Technology, Jiangxi Agricultural University, Nanchang, Jiangxi 330045, China; b Key Laboratory of Green and Healthy Breeding of Livestock and Poultry of Jiangxi Province, Institute of Animal Husbandry and Veterinary Medicine, Jiangxi Academy of Agricultural Sciences, Nanchang, Jiangxi 330200, China; c Jiangxi Provincial Engineering Technology Center for Animal Disease Prevention and Control Agents, Nanchang, Jiangxi 330045, China; d Key Laboratory of Jiangsu Preventive Veterinary Medicine, Key Laboratory for Avian Preventive Medicine, Ministry of Education, College of Veterinary Medicine, Yangzhou University, Yangzhou, Jiangsu 225009, China

**Keywords:** Goose astrovirus, Loop-mediated isothermal amplification, *Pyrococcus furiosus* argonaute, detection

## Abstract

Goose astrovirus (GAstV) is a newly emerged viral pathogen in goose, characterized by a high incidence and significant mortality rates. This etiology has repeatedly occurred in coastal areas of China, rapidly spreading to inland provinces in recent decade, and thus imposed huge economic losses on China’s goose industry. Therefore, it is essential to establish a rapid, accurate and sensitive method for GAstV diagnosis. In this study, we employed reverse transcription loop-mediated isothermal amplification (RT-LAMP) assay combined with a *Pyrococcus furiosus* Argonaute (*Pf*Ago) system, providing a simple and precise approach for GAstV. Specific primers and guide DNA (gDNA) were designed to target conserved regions of the GAstV genome. The assay achieved a detection limit of 10 copies/μL in preliminary validation assays when targeting conserved regions of the viral genome with optimized reaction conditions. Importantly, the assay exhibited no cross-reactivity with other viruses, including Goose parvovirus (GPV), Goose circovirus (GoCV), Tembusu Virus (TMUV), Muscovy Duck Reovirus (MDRV), and *Escherichia coli* (*E. coli*). Detection results from 59 clinical samples demonstrated complete concordance in positive rates between the LAMP-*Pf*Ago and qPCR methods.

## Introduction

GAstV is a newly emerged viral pathogen that causes novel gosling gout disease with two established genotypes (GAstV-I and GAstV-II) (Xu et al., 2023; Zhang et al., 2022). GAstV is the pathogen responsible for novel gosling gout disease, characterized by urate deposition in internal organs and joints, presenting typical gout symptoms (Wang et al., 2022). Current epidemiological data confirm GAstV-II as the predominant etiological agent responsible for gout outbreaks in geese (Zhu and Sun, 2022). GAstVs are non-enveloped viruses with a positive-sense, single-stranded RNA genome of 7.1 to 7.3 Kb (Yuan et al., 2019), which includes a 5′-untranslated region (UTR), three open reading frames (ORFs; ORF1a, ORF1b, and ORF2), a 3′UTR, and a poly(A) tail (Zhang et al., 2018a). ORF1a and ORF1b are highly conserved sequences encoding important non-structural proteins (NSPs) involved in viral replication and transcription (Cortez et al., 2017; Fu et al., 2022).

Since 2017, outbreaks of gout associated with goose astrovirus infection have been reported across China. This virus primarily affects goslings within 20 days of age, leading to growth retardation, younger goslings exhibiting higher mortality (An et al., 2020). The disease has spread rapidly nationwide, causing substantial economic losses (Wang et al., 2021). To date, studies on GAstV remain preliminary, and there is no effective treatment or vaccine. The primary focus of disease management is on outbreak surveillance and containment. Therefore, rapid diagnosis is critical for early surveillance and control of GAstV outbreak risks.

Diagnostic approaches for GAstV include virus isolation, enzyme-linked immunosorbent assay (ELISA), reverse transcription-polymerase chain reaction (RT-PCR), reverse transcription-quantitative PCR (RT-qPCR), RT-LAMP, and combined reverse transcription-enzymatic recombinase amplification (RT-ERA) with the Cas12a. Zhang et al. first discovered that GAstV could be isolated and propagated in LMH cell, a chicken liver cell line (Zhang et al., 2018b). However, isolating GAstV is difficult and time-consuming. Serological methods are fast and easy to operate but cannot timely and accurately reflect GAstV infection (He et al., 2023; Zhang et al., 2023). RT-PCR and RT-qPCR have been established for the detection of GAstV, usually with good accuracy and specificity, but they require cumbersome and precise equipment (Liu et al., 2025a; Yi et al., 2022; Yin et al., 2020). Furthermore, in recent years, with rapid advances in isothermal amplification technologies, such as LAMP, recombinase polymerase amplification (RPA), and recombinase-aided amplification (RAA) have been employed for the detection of various pathogens (Fan et al., 2020; Mao et al., 2022; Yu et al., 2019). However, the result is limited by non-specific amplification and false-positive. Currently, CRISPR-based rapid detection methods have been developed to mitigate false-positive results (Li et al., 2018; Liu et al., 2023; Yang et al., 2022). However, their application remains constrained by the requirement for protospacer adjacent motif (PAM) sequences adjacent to the target sites.

Argonaute (Ago)-based detection methods are increasingly recognized as a promising platform (Kropocheva et al., 2022). These methods activate nucleases through nucleic acid targeting, generating detection signals with high specificity without being limited by specific target sequences (Zhao et al., 2024). *Pf*Ago is a prokaryotic argonaute (pAgo) from *Pyrococcus furiosus*, is commonly employed for nucleic acid detection (Wang et al., 2023; Ye et al., 2022). In this study, a rapid detection method for GAstV was established by integrating LAMP and *Pf*Ago protein technologies. This method can effectively reduce the false positives by the specifically recognition and cleavage of LAMP products via *Pf*Ago, and improve the detection limit of *Pf*Ago through isothermal amplification (Xun et al., 2021; Yu et al., 2025). In the LAMP-*Pf*Ago analytical method established in this research, *Pf*Ago is a prokaryotic Argonaute (pAgo) from *Pyrococcus furiosus* (Swarts et al., 2015). As a nucleic-acid-guided endonuclease, it specifically cleaves the target between the 10th and 11th nucleotide bases from the 5′ to 3′ ends of aligned gDNA under the guidance of three short 5′-phosphorylated single-strand DNA, and producing a 16 nucleotide 5′-phosphorylated single-stranded DNA (ssDNA) fragment. The cleavage generates a ssDNA fragment that functions as a secondary gDNA to guide *Pf*Ago for secondary cleavage, leading to the split of the quenchers from the fluorophore (He et al., 2019). Finally, the fluorescence signal can be detected with a real-time fluorescent qPCR detector or fluorescence spectrometer. Probe and gDNA sequences were designed between the FIP and BIP of the LAMP primer. This design ensures that the sequences recognized by *Pf*Ago are derived from the amplification of taret sequences rather than non-specific amplification in the LAMP reaction. To prevent aerosol contamination during the reaction, the LAMP amplification system was added to the bottom of the reaction tube, and the *Pf*Ago cleavage system was added to the lid of the tube. After the completion of the LAMP amplification reaction, a simple centrifugation step was performed to mix the LAMP and *Pf*Ago detection systems, and the results can be visualized under UV light. Therefore, the single-tube LAMP-*Pf*Ago detection platform minimizes aerosol contamination and enables rapid and sensitive detection of GAstV. In this study, we developed a single-tube assay for detecting GAstV that integrates LAMP with the *Pf*Ago system. This method is completed within 60 minutes with high sensitivity and specificity, providing a rapid, reliable and convenient method for GAstV detection.

## Materials and methods

### Ethics statement

All samples were collected on commercial goose farms by veterinarians during routine diagnostic sampling after permission from the farm owner. No specific permits from an animal ethics committee were required.

### viruses and clinical samples

The GAstV strain JXGZ/2021 (GenBank accession number: OL762473) was provided by the Institute of Animal Husbandry and Veterinary Medicine, Jiangxi Academy of Agricultural Sciences. Viruses isolated in this study were archived at the Institute of Animal Husbandry and Veterinary Medicine, Jiangxi Academy of Agricultural Sciences, including: GPV, GoCV, TMUV, and E. coli. Viral genomic RNA was extracted using RNAiso Plus (Takara, Beijing, China) according to the manufacturer's instructions, and cDNA synthesis was performed with PrimeScript RT Master Mix (Takara, Beijing, China). The resulting cDNA/DNA templates were stored at −80°C until further use.

### Construction of recombinant plasmid

The target fragments were amplified by PCR, purified using a Gel Extraction Kit (Omega Bio-tek, Guangzhou, China), and cloned into the pMD-19T vector (Takara, Beijing, China). The recombinant plasmids were transformed into Top10 competent cells (Tolo Bio, Hong Kong, China). DNA sequencing was performed by Sangon Biotech (Shanghai, China). Plasmid extraction used with a TIAN prep Mini Plasmid Kit (TIANGEN, Beijing, China). The concentration was measured on a NanoDrop 2000 spectrophotometer (Thermo Fisher Scientific, Waltham, MA, USA). The plasmid copy number was calculated using the following formula: copies/μL = (6.02 × 10^23^) × (ng/μL × 10^-9^) / (length of DNA × 660). Serial 10-fold dilutions were performed to obtain plasmid concentrations ranging from 1 × 10^6^ to 1 × 100 copies/μL.

### Primer design

The conserved ORF1b gene region of GAstV was targeted to design LAMP primers using the online primer software Primer Explorer V5 (https://primerexplorer.eiken.co.jp/e/), including an external primer pair (F3 and B3), an internal primer pair (FIP and BIP), and two loop primers (LB and LF). Additionally, three gDNA sequence and corresponding probes were designed based on PfAgo cleavage specificity, the probes were modified with FAM, and the 5′ ends of the gDNA were phosphorylated. The primers qPCR-F and qPCR-R were utilized for qPCR targeting GAstV (Xu, 2019). The designed primers, gDNAs, and probes were synthesized by Sangon Biotech (shanghai China). The sequence information for the primers and gDNAs is listed in Table 1.

**Table 1:** Sequences of primers, gDNAs and probe used in this study.

Label	Sequence (5′−3′)
F3	GCAGGACCAGAATGAGAA
B3	TCTTATGATGGTTGGACAGGAA
FIP	TCCACCAAAAAAGGGTGTCCATGAAGCAACAGACAGAACG
BIP	ACAGGTTTTTTGTAGAGACGGATTCGCATCTGTCGTATYCGC
LF	CAACTTGTGCAGCCCGC
LB	GGACGCGTTATGATGGTACG
gDNA1	GAATACATCAGCGAGT
gDNA2	AGATACTCGCTGATGT
gDNA3	CACCACCAATGAGCCT
Probe	FAM-GAGCCTAGATACTCGCT-BHQ1
qPCR-F	TGAAGCAACAGACAGAACGG
qPCR-R	GGACAGGAAAAAGTAACGCA

### Optimization of LAMP reaction conditions

LAMP reactions were performed following the manufacturer's protocol for Bst 2.0 WarmStart DNA Polymerase (New England Biolabs, Ipswich, MA, USA). Each 25 μL reaction mixture contained 2.5 μL of 10 × Isothermal Amplification Buffer, 6 mM MgSO₄, 1.4 mM dNTP mix (Takara, Beijing, China), 1.6 μM internal primer (FIP/BIP), 0.2 μM external primers (F3/B3), 0.4 μM loop primers (LF/LB), 8 U Bst 2.0 WarmStart DNA Polymerase, and 1 μL template DNA, with nuclease-free water added to achieve a final volume of 25 μL. Reactions were incubated at 65°C for 30 min in a thermal cycler or isothermal heating device. Amplification products were electrophoresed on 1.5% agarose gels in 1 × TAE buffer or directly visualized by adding 1 × SYBR Green I in the reaction tube for diction by a color change. To achieve the best reaction conditions, a systematic optimization process was carried out by adjusting the concentration of MgSO₄(2, 4, 6, 8, 10, 12 mM), the concentration of dNTPs (0.2, 0.4, 0.6, 0.8, 1.0, 1.2, 1.4 mM), internal and external primer concentration ratio (1:1, 4:1, 8:1, 12:1, 14:1), reaction temperature (53, 57, 61, 65, 69°C), and reaction time (10, 20, 30, 40, 50, 60 min).

### Optimization of the LAMP-PfAgo reaction system

First, LAMP reaction mixture was dispensed into the tube bottoms. Then PfAgo system was added, which included 2 μL of 10 × reaction buffer, 1.0 mM Mn²⁺, 2.0 μM of each gDNA, and 3 μL PfAgo (200 U/μL), and tube caps were affixed to filter paper discs preloaded with 0.25 μM probes. To immerse discs, tubes were centrifuged at 5,000 × *g* for 1 min after incubation for 30 minutes at 65°C. Tubes were mixed and incubated for 30 min at 95°C. The reaction results were observed in a fluorescence detector or under UV light. To achieve the best reaction conditions, a systematic optimization process was carried out by adjusting the concentration of gDNA (1, 3, 5, 7, 10 μM), MnCl (0.5, 1.0, 1.5, 2.0, 3.0 μM), and PfAgo enzyme (200, 400, 600, 800, 1000 U).

### LAMP-PfAgo sensitivity and specificity tests

To evaluate the sensitivity, serially diluted plasmids were used as templates to determine detection limit of the LAMP-PfAgo assay in vitro. For specificity assay, six common pathogens were analyzed. The pathogens included GPV, GoCV, TMUV, MDRV, E. coli. Results were visualized under UV light or blue light conditions after 30 min LAMP-PfAgo reaction at 95°C.

### Clinical evaluation of LAMP-PfAgo method

To validate the LAMP-PfAgo assay for GAstV detection, 59 clinical intestinal samples from gout-afflicted geese were collected. All samples underwent concurrent analysis using both LAMP-PfAgo and qPCR for comparative evaluation.

### Statistical analysis

Schematic diagrams were created using Adobe Illustrator 2020 (Adobe Inc., San Jose, CA). Data were analyzed by one-way ANOVA using GraphPad Prism 5.0 (GraphPad Software, San Diego, CA). Values are presented as mean ± SEM. Experiments included three technical replicates. Statistical significance is indicated as follows: *P < 0.05, **P < 0.01, ***P < 0.001, and ****P < 0.0001.

## Results

### Mechanism of the LAMP-PfAgo detection method

**Fig. 1:**
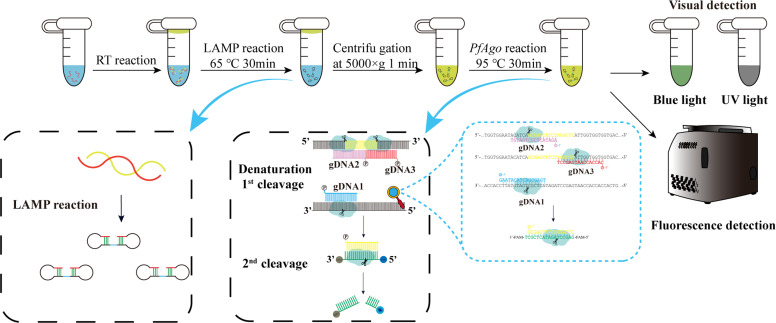
Workflow of the RT-LAMP-*Pf*Ago assay for GAstV detection.

Fig. 1 illustrates the workflow for detecting GAstV genomic RNA using the LAMP-PfAgo assay. Briefly, the LAMP amplicons are specifically identified by PfAgo protein under the guidance of 5′-phosphorylated guide DNA (gDNA). Following base pairing between the gDNA and one strand of the LAMP amplicon, PfAgo cleaves the phosphodiester bond between the 10th and 11th nucleotides of the target DNA, thereby generating a newly formed 5′-phosphorylated ssDNA. This ssDNA can subsequently serve as a gDNA to guide PfAgo to cleave the secondary cleavage molecular beacon probe, releasing the fluorescent groups. The fluorescent signal can be detected with a fluorescence detector or visualized under UV or blue light.

### Optimization of LAMP reaction

**Fig. 2:**
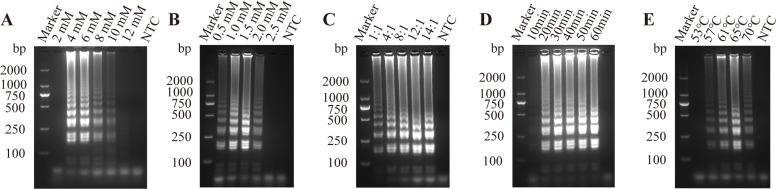
Optimization of LAMP reaction conditions. Screening for optimal concentrations of (A) MgSO_4_, (B) dNTPs, (C) internal and external primers for LAMP assay; (D) Screening for optimal reaction times and (E) temperature for LAMP assay.

To optimize the LAMP reaction system, the concentrations of key components were adjusted, including the concentration of MgSO4, the concentration of dNTPs, internal and external primer concentration ratio (Fig. 2A–C). To further enhance amplification efficiency, reaction time and temperature were optimized (Fig. 2D and E). Taken together, the optimal conditions were determined as follows: 6 mM MgSO4, 1.4 mM dNTP, an internal to external primer concentration ratio of 12:1, a reaction temperature of 65°C, and a reaction time of 30 min.

### Optimization of LAMP-PfAgo reaction

**Fig. 3:**
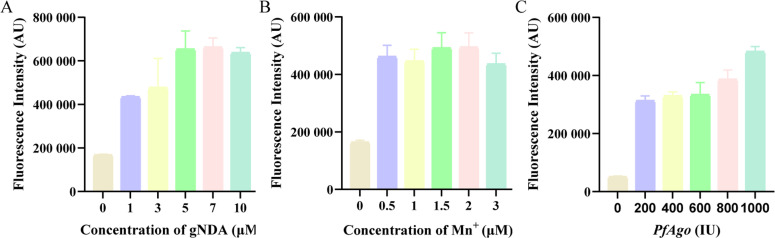
Optimization of components for the LAMP-*Pf*Ago assay. Fluorescence intensity of LAMP-*Pf*Ago reaction at different concentrations of (A) Mn^2+^, (B) *Pf*Ago, and (C) gDNA.

To enhance the LAMP-PfAgo reaction efficiency, the concentrations of key components were optimized, including MnCl₂, gDNA, and PfAgo (Fig. 3A and C). The optimal reaction conditions were determined as follows: 7 μM gDNA, 1.5 mM MnCl2, and 1000 U of PfAgo.

### Sensitivity and specificity of the LAMP-PfAgo

**Fig. 4:**
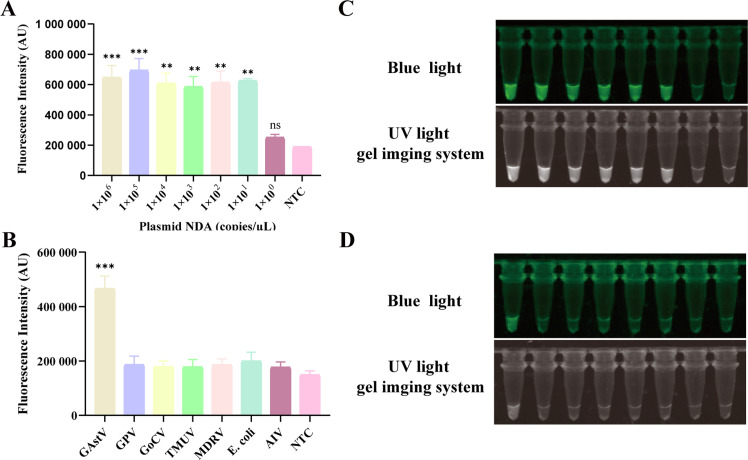
Evaluation of the sensitivity and specificity of LAMP-*Pf*Ago assay. Endpoint fluorescence values indicating LAMP-*Pf*Ago (A) sensitivity and (B) specificity; Images capture indicating (C) sensitivity and (D) specificity under UV light and blue light. Each experiment was performed in triplicate and statistical analysis was conducted using student’s *t*-test in GraphPad Prism 8. NTC indicates negative control; ** indicates *p* < 0.01; *** indicates *p* < 0.001; **** indicates *p* < 0.0001; ns indicates no significance.

To evaluate the sensitivity of the LAMP-PfAgo assay, the recombinant plasmid templates serially diluted from 1 × 106 to 1 × 100 copies/μL were tested to determine the detection limit. Results demonstrated a limit of detection (LOD) of 10 copies/μL (Fig. 4A), with visual detection under UV and blue light yielding consistent results (Fig. 4B). Specificity testing of the LAMP-PfAgo assay was performed using common clinical pathogens including GPV, GoCV, and TMUV. The assay produced fluorescence signals only when GAstV was detected, with no cross-reactivity detected with other pathogens (Fig. 4C), with visual detection under UV and blue light yielding consistent results (Fig. 4D).

### Clinical sample evaluation

**Fig. 5:**
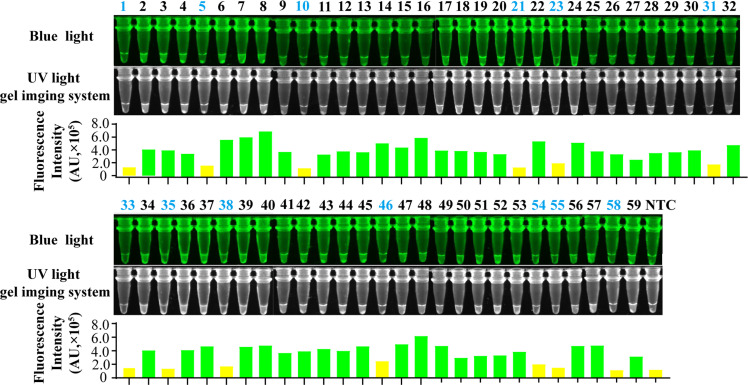
Reliability of the LAMP-*Pf*Ago assay for detecting clinical samples. Samples in colored in blue indicate a negative result. NTC indicates negative control.

To evaluate the clinical applicability of the LAMP-PfAgo assay, the clinical samples suspected of GAstV infection were tested using both RT-LAMP-PfAgo and qPCR assays. Both assays detected 40 positive samples and 30 negative samples. The images captured under UV or blue light were consistent with those of fluorescence detection (Fig. 5). These findings demonstrate that the LAMP-PfAgo method is suitable for the detection of GAstV in clinical diagnostic specimens.

## Discussion

Since 2016, a disease characterized primarily by urate deposition in visceral organs and joints has been observed in several regions of China, including Guangdong, Hunan, Shandong, Jiangsu, Fujian, and Henan (Wan et al., 2018). The pathogen predominantly affects goslings within 20 days of age, with mortality rates reaching up to 50%, causing significant economic losses to the poultry industry in China. Currently, there are no effective detection methods or preventive measures available. Moreover, as most farms are located in remote areas, the risk of disease transmission is indirectly increased. To date, several diagnostic methods have been developed for GAstV, including ELISA, RT-PCR, RT-qPCR, RT-LAMP, and RT-ERA-CRISPR/Cas12a.Although RT-PCR and RT-qPCR are widely used for viral detection, they rely on specialized equipment and involve complex procedures, limiting their applicability for clinical diagnosis. Compared with other rapid detection systems, such as RT-LAMP and RT-ERA-CRISPR/Cas12, standalone RT-LAMP can rapidly amplify nucleic acids, but the results are prone to false positive results due to non-specific amplification. Although RT-ERA-CRISPR/Cas12a system has high sensitivity, it si limited by the high synthetic cost of gDNA and the dependence on the PAM. Therefore, the development of a rapid, accurate, sensitive, portable, and cost-effective diagnostic technology is urgently needed to enable efficient on-site screening.

*Pf*Ago can bind to short gDNA molecules for specific target recognition and exhibits the ability of precise guided cleavage (Hong et al., 2025; Swarts et al., 2015). This capability ensures the targeted elimination of non-specific amplification products, significantly reducing the possibility of false-positive results, providing a new platform for nucleic acid molecular diagnosis, and demonstrating broad application potential (Wu et al., 2023). In recent years, the combined application of *Pf*Ago protein and isothermal amplification technology has pioneered a detection method with high sensitivity, high specificity, and rapidity in the field of nucleic acid detection, demonstrating tremendous advantages in on-site rapid diagnosis (Liu et al., 2024, 2025b; Yu et al., 2024).We combine *Pf*Ago with LAMP to increase the convenience and specificity of detection based on the high sensitivity and specificity of *Pf*Ago cleavage activity, and it can effectively prevent false positive results caused by the amplification process, thereby ensuring the accuracy of the diagnostic results because of its stepwise cleavage specificity. This method was further validated with 30 clinical samples. Our findings demonstrated that the RT-LAMP-*pf*Ago based assay yielded identical results to those obtained from RT-qPCR tests, indicating that its reliability was comparable to that of the current gold standard. Additionally, the RT-LAMP-*pf*Ago assay is faster and involves a simpler workflow compared to qPCR.

In summary, the RT-LAMP-*pf*Ago assay developed in the study can detect GAstV, and offers advantages including operational simplicity, rapidity, and high specificity. The assay is applicable for GAstV detection in epidemiological surveys.

## Conclusions

In summary, we have successfully developed a rapid and highly sensitive detection method for detecting GAstV by integrating LAMP with *Pf*Ago. The sensitivity of this LAMP-*Pf*Ago assay is 10 copies/µL, and it does not cross-react with other pathogens. Furthermore, the procedure is straightforward does not require precision instruments, making it suitable for the clinical detection of GAstV.

## Disclosures

Author Baobao Xie is employed by Dabeinong Technology Co.,Ltd. of Jiangxi. Author Yangyang Luo is employed by Wen's Foodstuff Group Co., Ltd., Wen's Group Research Institute. The remaining authors declare that the research was conducted in the absence of any commercial or financial relationships that could be construed as a potential conflict of interest.

## Data availability statement

The data that support the findings of this study are available from the corresponding authors upon reasonable request.

## CRediT authorship contribution statement

**Fosheng Yang:** Writing – original draft, Software, Methodology, Data curation. **Zhong Liu:** Validation, Supervision, Methodology, Formal analysis. **Jianqiang Ye:** Writing – review & editing, Writing – original draft, Funding acquisition. **Quan Xie:** Resources, Project administration, Methodology, Investigation. **Chang Wu:** Methodology, Investigation, Formal analysis. **Chengjun Jiang:** Validation, Data curation, Conceptualization. **Huangsheng Wu:** Resources, Project administration. **Qianlang Gu:** Validation, Supervision, Methodology, Conceptualization. **Deping Song:** Writing – review & editing, Writing – original draft. **Fanfan Zhang:** Writing – review & editing, Resources, Methodology, Funding acquisition.
